# Matrix degradability controls multicellularity of 3D cell migration

**DOI:** 10.1038/s41467-017-00418-6

**Published:** 2017-08-29

**Authors:** Britta Trappmann, Brendon M. Baker, William J. Polacheck, Colin K. Choi, Jason A. Burdick, Christopher S. Chen

**Affiliations:** 1 The Biological Design Center and Department of Biomedical Engineering, Boston University, Boston, MA 02215 USA; 2 Wyss Institute for Biologically Inspired Engineering, Harvard University, Boston, MA 02115 USA; 3 Department of Bioengineering, University of Pennsylvania, Philadelphia, PA 19104 USA

## Abstract

A major challenge in tissue engineering is the development of materials that can support angiogenesis, wherein endothelial cells from existing vasculature invade the surrounding matrix to form new vascular structures. To identify material properties that impact angiogenesis, here we have developed an in vitro model whereby molded tubular channels inside a synthetic hydrogel are seeded with endothelial cells and subjected to chemokine gradients within a microfluidic device. To accomplish precision molding of hydrogels and successful integration with microfluidics, we developed a class of hydrogels that could be macromolded and micromolded with high shape and size fidelity by eliminating swelling after polymerization. Using this material, we demonstrate that matrix degradability switches three-dimensional endothelial cell invasion between two distinct modes: single-cell migration and the multicellular, strand-like invasion required for angiogenesis. The ability to incorporate these tunable hydrogels into geometrically constrained settings will enable a wide range of previously inaccessible biomedical applications.

## Abstract (web summary)

The fabrication of vascularized 3D tissues requires an understanding of how material properties govern endothelial cell invasion into the surrounding matrix. Here the authors integrate a non-swelling synthetic hydrogel with a microfluidic device to study chemokine gradient-driven angiogenic sprouting and find that matrix degradability modulates the collectivity of cell migration.

## Introduction

Synthetic hydrogels are widely used as models of the extracellular matrix (ECM) and have been instrumental in developing our understanding of how physical and adhesive properties of the ECM can regulate cell function^1–3^. Within organs, the architecture of the ECM organizes and segregates cell populations, and defines fluid vs. solid domains (e.g., vascular vs. stromal spaces). To recapitulate such features, whether for “on-chip” models of organs or for engineered tissues for replacement therapies, synthetic hydrogels amenable to precision molding could aid in accurately defining architectures found in biological tissues. However, most existing synthetic hydrogel systems are difficult to precision mold due to the swelling of the material that occurs upon equilibrium hydration^4^. Such swelling not only alters the gross geometry and dimensions of the initially defined structure, but also changes the mechanical properties^5^ and nanoscale structure of these materials.

Equilibrium swelling of hydrogels occurs when elastic retractive forces of the polymer network are balanced by attractive forces between the polymer chains and water, which increase with polymer hydrophilicity^4^. We hypothesized that increasing the hydrophobicity of the polymer backbone through the attachment of hydrophobic pendant side chains could modulate and possibly eliminate swelling, thereby generating hydrogels amenable to precision molding. We illustrate the utility of this system in several settings, and through integration with a microfluidic device, reveal that endothelial cell migration into the surrounding 3D matrix adopts two distinct modes as a function of matrix degradability.

## Results

### Design of non-swelling hydrogels via tuning hydrophobicity

**Fig. 1:**
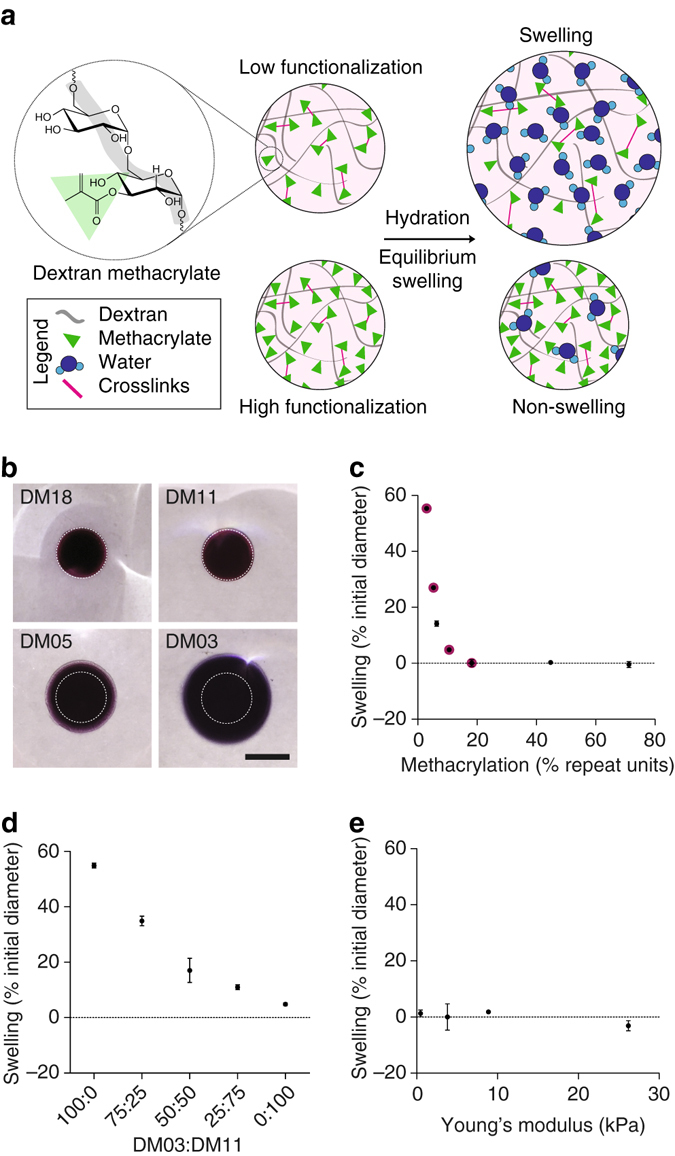
Design of a class of hydrogels with controlled swelling properties. **a** Schematic representation of DexMA hydrogels with controlled swelling properties via addition of hydrophobic methacrylates. **b** UV-crosslinked DexMA hydrogels with different degrees of methacrylation (label indicates % methacrylation), corresponding to the *magneta*-labelled data points in **c** after equilibrium swelling. Initial dimensions immediately following crosslinking are outlined by dotted lines (*scale bar*, 500 μm). **c** Swelling (% change in gel diameter relative to initial diameter before equilibrium hydration) as a function of dextran backbone methacrylation. **d** Swelling of UV-crosslinked hydrogels made of DexMA with 3 and 11% methacrylation mixed at various ratios. **e** Swelling as a function of the mechanical properties (Young’s modulus in kPa) of the gels. All data is presented as a mean ± s.d

To develop a cytocompatible hydrogel with controlled swelling upon equilibrium hydration, we chose methacrylated dextran (DexMA)^6–8^ as a base material that is biologically inert, in that it resists protein adsorption and has no known cell surface receptor binding activity (Fig. 1a). DexMA macromers were crosslinked through Michael-type addition with matrix metalloproteinase (MMP) labile dicysteine peptide sequences (Fig. 1a), a strategy that has been demonstrated with other backbones^9, 10^. We speculated that increasing the hydrophobicity of the dextran polymer chains would result in a reproducible reduction in swelling of the resulting hydrogels, and that we could achieve this tuning through the attachment of increasing amounts of intrinsically hydrophobic methacrylates to the dextran backbone prior to crosslinking (Supplementary Fig. 1). Indeed, when we gradually increased the level of methacrylate functionalization from 3 to 70% (at higher methacrylations, DexMA was no longer water soluble), hydrogel swelling (final gel volume normalized to initial volume) decreased from 55 to 0% (Fig. 1b, c and Supplementary Fig. 2). In addition to individual macromer hydrophobicity, we hypothesized that the aggregate hydrophobicity defined by mixtures of dextrans of varying hydrophobicities would also dictate swelling. Indeed, mixing DexMA with higher (11%) and lower (3%) levels of methacrylation at different ratios (Fig. 1d) produced hydrogels that swelled according to the average methacrylation levels of the macromers. Thus, hydrogel swelling behavior appears to be defined by the overall hydrophobicity of the component mixture, even if the individual monomer units are distinct. An important outcome of the approach is that the degree of swelling is not affected by the amount of crosslinking introduced to the polymer networks, allowing swelling to be controlled independently of hydrogel stiffness (Fig. 1e).

### Multi-scale molding applications for non-swelling hydrogels

**Fig. 2:**
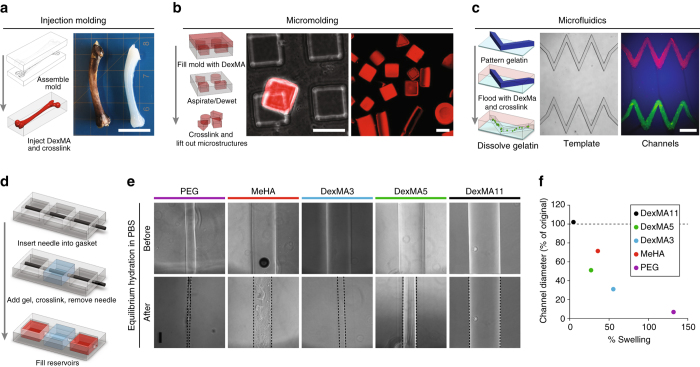
Multi-scale molding and microfluidic integration of non-swelling DexMA. **a** Replica-molding of DexMA to duplicate the gross anatomical features of a bone. A negative mold of the bone was generated in PDMS, filled with DexMA gel precursor solution, and UV crosslinked to yield the bone replicate (*scale bar*, 1 cm). **b** DexMA micro-gels were similarly fabricated by replica-molding using PDMS molds generated by traditional SU8 photolithography (*scale bars*, 100 μm). **c** Patent micron-scale fluidic channels embedded within non-swelling DexMA gels. Sacrificial channel structures were micromolded in gelatin as in ref. ^32^, DexMA gels were cast on top, and gelatin was dissolved at 37 °C to yield open channels. Brightfield and fluorescence image demonstrating channel perfusion by red and green fluorescent beads (*scale bar*, 500 μm). **d** Schematic depicting fabrication of tubular channels embedded within 3D hydrogels. **e** Images of channels immediately after needle removal, and following equilibrium hydration. Swelling hydrogels (DexMA3, DexMA5, MeHA, PEG) all result in channel closure in contrast to non-swelling DexMA11. *Scale bar*, 100 µm. **f** Degree of channel closure (% of initial channel width) as a function of hydrogel swelling (defined as % of initial gel diameter)

Next, we demonstrated the ability of this non-swelling hydrogel to define and maintain precise geometric structures. Injection molding of DexMA recreated the complex macroscopic anatomic features of a femur (Fig. 2a). To examine whether geometric features even at the micrometer scale could be prescribed, we micromolded DexMA hydrogels into photolithographically defined molds of various geometries. The resultant microgels matched the micrometer scale dimensions and shapes of the molds from which they were cast (Fig. 2b). In addition to the generation of free-standing constructs with well-defined shapes and dimensions, non-swelling materials are critical for integrating hydrogels into multi-component devices, such as lab-on-chip systems. To illustrate, we show how non-swelling DexMA hydrogels can be cast to form well-defined microfluidic channels within a PDMS casing that integrates microfluidic ports (Fig. 2d). To examine the impact of swelling on the fidelity of precision molding, we generated channel structures inside dextran and non-dextran-based hydrogels of varying hydrophobicities. Results show that the channel diameters shrink upon equilibrium hydration as a function of hydrogel swelling, and only non-swelling DexMA gels are able to maintain the predetermined channel diameter (Fig. 2e, f). While the impact of swelling on simple cylindrical channels is merely changes in channel diameter, faithful fabrication of more complex features such as channels with sharp turns requires non-swelling materials (Fig. 2c).

### Integrating hydrogels with microfluidics to study angiogenesis

**Fig. 3:**
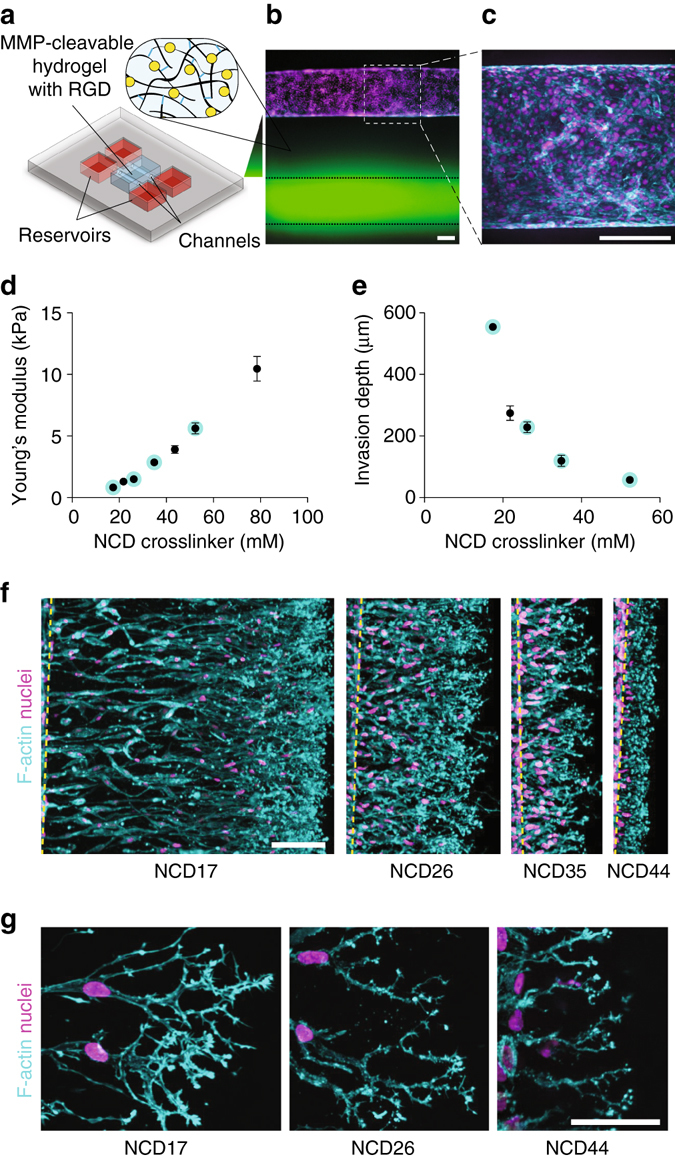
Angiogenic sprouting is influenced by matrix crosslinking. A microfluidic platform is employed to study endothelial cell sprouting from a parent channel into surrounding DexMA matrices of varying MMP-labile crosslinker concentrations. **a** Schematic of the microfluidic device employed in these studies. **b** The device consists of two parallel channels fully embedded within a DexMA gel. A growth factor cocktail is added to one channel, creating a chemoattractive gradient (*green arrow*). **c** The other channel is seeded with human umbilical cord vein endothelial cells (HUVECs). **d** Young’s modulus of DexMA hydrogels with varying concentrations of native collagen degradability (NCD) crosslinker, corresponding to samples shown in (**e**). **e** Quantification of HUVEC invasion depth as a function of NCD crosslinker concentration. **f** HUVECs invading into DexMA gels of different crosslinker concentrations (indicated by *cyan*-labeled data points in **e**, **f**) 48 h after growth factor cocktail addition. Composite fluorescence images showing F-actin (*cyan*) and nuclei (*magenta*). *Dashed yellow lines* indicate HUVEC channel position (*scale bar*, 100 μm). **g** Morphology of tip cells invading into DexMA gels of varying crosslinker concentrations. Composite fluorescence images showing F-actin (*cyan*) and nuclei (*magenta*) (*scale bar*, 50 μm). All data are presented as a mean ± s.d.

This ability to integrate non-swelling DexMA hydrogels into microfluidic devices, together with the ability to incorporate other bioactive features essential to controlling cell behavior (e.g., cell adhesiveness and tunable mechanical properties (Supplementary Fig. 3a, b)), provides an ideal system for investigating how matrix properties regulate cellular behaviors observed most readily in microfluidic systems. To explore this utility, we incorporated these gels into a previously developed microfluidic platform used to recapitulate angiogenesis, as understanding how biomaterial properties can be tuned to promote angiogenesis would enable numerous regenerative medicine applications^11^. In this system, angiogenic sprouting occurs from a perfused endothelial cell-lined channel triggered by pro-angiogenic soluble gradients across a collagen matrix^12^ (Fig. 3a); earlier attempts to incorporate synthetic hydrogels were thwarted by hydrogel swelling and subsequent channel collapse (Fig. 2e, f). To generate the endothelial cell-lined channel, one cylindrical channel passing through a non-swelling MMP-degradable DexMA gel (Fig. 3a, b) was seeded with endothelial cells to form a confluent endothelium serving as a parent vessel (Fig. 3c). Introducing a cocktail of angiogenic growth factors in a second parallel channel under continuous flow created a chemoattractive gradient (Supplementary Fig. 4) and triggered multicellular sprouting. We then used this system to ask whether the sprouting response was affected by changing the stiffness of the matrix, as matrix stiffness has previously been described to modulate a number of cell functions including proliferation, differentiation, and migration^13–15^. Holding the degree of dextran methacrylation constant (70% of repeat units contained a methacrylate group), we tuned hydrogel stiffness via the concentration of MMP labile dicysteine peptides, thereby modulating the number of resulting crosslinks within the hydrogel network (Fig. 3d). Despite increased hydrophobicity via methacrylation, these gels did not exhibit non-specific cell adhesion over the full range of crosslinking explored in these studies, as serum-exposed gels did not support cell adhesion without RGD coupling. Increased stiffness not only decreased sprout length (Fig. 3e, f), but also altered the morphology of the leading tip cell, from very open, branched structures with long filopodia in lightly crosslinked matrices, to narrow branches with short and spiky filopodia in highly crosslinked DexMA gels (Fig. 3g).

### Matrix crosslinking influences angiogenic sprouting

**Fig. 4:**
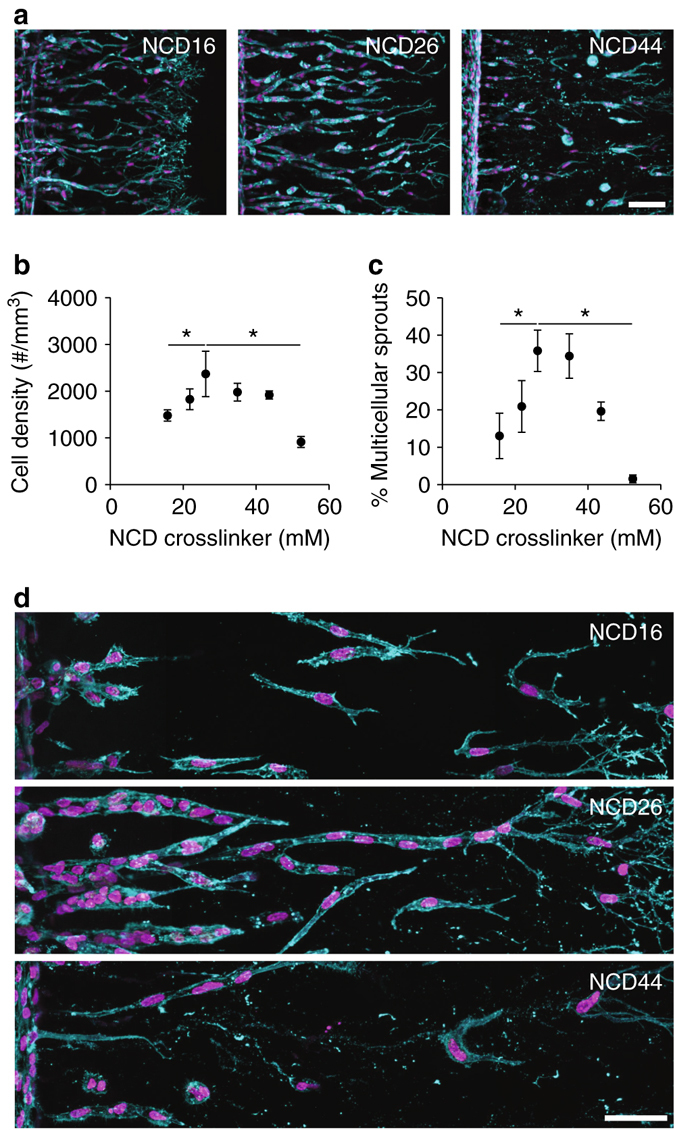
Angiogenic sprout multicellularity is controlled by matrix crosslinking. **a** HUVECs invading into DexMA gels crosslinked with 16, 26, and 44 mM MMP-labile NCD peptides analyzed at a constant invasion depth after 1, 3.5, and 15 d, respectively. Composite fluorescence images showing F-actin (*cyan*) and nuclei (*magenta*) (*scale bar*, 100 μm). **b** Quantification of HUVEC density as a function of NCD crosslinker concentration. **c** Sprout multicellularity (% of sprouts possessing six or more nuclei) as a function of NCD crosslinker concentration. **d** Morphology of sprouts invading into DexMA gels of different NCD crosslinker concentrations. Composite fluorescence images showing F-actin (*cyan*) and nuclei (*magenta*) (*scale bar*, 50 μm). All data are presented as a mean ± s.d., *P < 0.05

While increasing crosslinking density would be expected to limit the extent of invasion, an unexpected change occurred in the critical ability of cells to sprout collectively as multicellular strands. We observed a switch from highly collective multicellular sprouting in gels with intermediate crosslinking, to less coordinated migration involving single or small groups of cells invading in lightly crosslinked gels. However, given the substantially deeper invasion of cells in lightly crosslinked gels that may lead to variations in chemokine concentrations experienced by the tip cells, it was difficult to directly attribute changes in sprout multicellularity to changes in matrix properties per se. That is, the loss of connectivity among endothelial cells was most dramatic in the samples where cells invaded to the greatest extent, suggesting the possibility that the slower invading cells in stiffer gels would dissociate when given additional time to invade further. To address this possibility, we repeated the study but fixed samples at different time points when cells and sprouts reached the same invasion depth (Fig. 4a). Even when controlling for invasion depth, cells primarily invaded alone into matrices of low crosslinking density, whereas intermediate crosslinking densities gave rise to multicellular sprouts and a higher cell density (Fig. 4b–d and Supplementary Figs. 5 and 6). Interestingly, allowing sufficient time for cells to invade substantially into highly crosslinked hydrogels again revealed single-cell migration, showing that the collective mode of invasion appears to be biphasic with respect to matrix crosslinking density (Fig. 4b–d and Supplementary Figs. 5 and 6). To exclude the possibility that varying crosslinking density alters the chemokine gradient profile, which could directly alter sprout morphology, we characterized the diffusivity and hydraulic permeability of hydrogels with low and intermediate crosslinking and found comparable values (Supplementary Table 1). In addition, we eliminated the possibility that cell proliferation contributed to multicellular sprout formation, as proliferation determined by EdU incorporation was equivalently negligible in all hydrogel conditions (Supplementary Fig. 8).

### ECM degradability dictates angiogenic sprout multicellularity

**Fig. 5:**
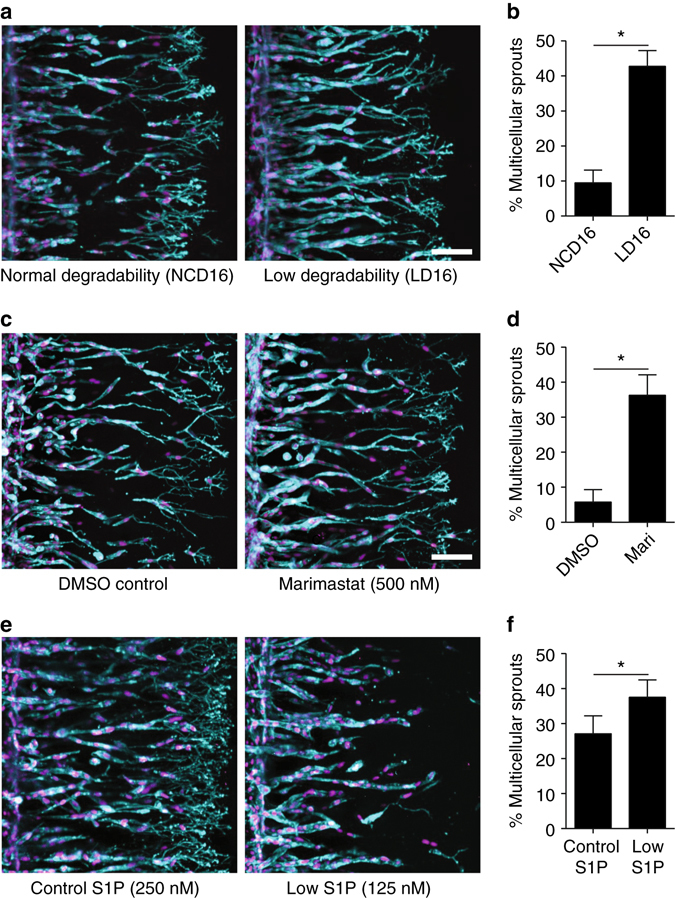
Matrix degradability controls angiogenic sprout multicellularity. **a** HUVECs invading into DexMA gels crosslinked with NCD and LD peptide sequences. Crosslinker concentration was kept constant at 16 mM to ensure comparable gel stiffness (1000 Pa). **b** Sprout multicellularity of DexMA gels crosslinked with NCD and LD sequence. **c** HUVECs invading into soft (16 mM NCD crosslinker) DexMA gels in the presence of a broad spectrum MMP inhibitor, Marimastat, or DMSO carrier only. **d** Sprout multicellularity with and without Marimastat treatment. **e** HUVECs invading into intermediate (26 mM NCD crosslinker) DexMA gels with varying S1P concentration. **f** Sprout multicellularity as a function of S1P concentration. **a**, **c**, and **e** contain composite fluorescence images showing F-actin (*cyan*) and nuclei (*magenta*) (*scale bar*, 100 μm). The % of sprouts possessing six or more nuclei was used as a metric for sprout multicellularity in (**b**, **d**, **f**), and all data are presented as a mean ± s.d. and significance was determined from a *P < 0.05

In the context of 3D culture, changes in crosslinking density not only impact matrix stiffness, but also how rapidly cell-mediated degradation of the hydrogel yields open space required for cell spreading, migration, and angiogenic sprouting. Hydrogel degradability, or the rate at which cells solubilize a given volume of hydrogel, is a function of the susceptibility of crosslinks to proteolytic cleavage and the number of crosslinks present. Thus, although one possibility is that the stiffness of the surrounding matrix influenced cell–cell adhesion^16^ triggering the switch between collective and single cell migration observed in our studies, another explanation is that the higher degradation rate of gels at lower crosslinking densities accelerated cell migration and enabled cells to break cell–cell junctions during migration. To tease apart the relative contributions of matrix stiffness vs. gel degradation rate in the observed differences in migration mode, we prepared hydrogels with a stiffness of 1000 Pa (a value correlated with single-cell migration in earlier studies) while modulating the susceptibility of the crosslinker sequence to MMP cleavage in order to modulate the degradation rate of the gel. This was achieved by replacing the standard sequence taken from the cleavage site of natural collagen^17^ (termed NCD for native collagen degradability) used in the above studies with a similar sequence containing a single amino acid mismatch that lowers MMP binding affinity^17^ (termed LD for lower degradability sequence) (Supplementary Fig. 3c). As anticipated, lowering crosslink susceptibility to MMP cleavage reduced the invasion speed of the sprouts (Supplementary Fig. 7). Strikingly, the transition to non-collective migration that occurs in lightly crosslinked matrices using NCD was reversed when using the LD crosslinker sequence. That is, collective invasion was rescued by lowering the rate of degradation of the hydrogel, suggesting that the mechanism by which collective migration is lost or maintained is due to matrix degradability rather than matrix stiffness (Fig. 5a, b).

To provide further support for this mechanism, we confirmed that rapid MMP-mediated degradation of the surrounding hydrogel matrix caused cells to invade as single cells. Rather than vary the crosslinker sequence, which could potentially introduce unintended biological activity in cellular interactions, we directly curbed enzymatic activity of cellular MMPs through pharmacologic means. Exposure to Marimastat, a broad spectrum MMP inhibitor, slowed cell invasion, and again rescued multicellular sprout formation in soft gels crosslinked with the NCD sequence, confirming the important interaction between cell-generated proteases and hydrogel degradation rate in multicellular invasion and sprout morphogenesis (Fig. 5c, d). Lastly, to determine whether invasion speed itself was a critical determinant in this response, we lowered the concentration of chemoattractant in the angiogenic growth factor cocktail to slow cell invasion. Halving the concentration of sphingosine-1-phosphate (S1P, 125 nM) directly slowed invasion speed without altering the hydrogel matrix or cellular MMP activity, and led to an enhancement in the number of multicellular sprouts invading into NCD-crosslinked gels as compared to the standard S1P concentration (250 nM) (Fig. 5e, f). Taken together, these studies provide evidence for a relationship between matrix crosslinking density and degradability on the one hand and the degradative activity of cell-produced MMPs on the other, the balance of which regulates 3D invasion speed and toggles cells between single and multicellular modes of migration.

## Discussion

Numerous studies using natural matrices such as fibrin or collagen have suggested that the physical properties of the ECM can regulate angiogenic sprouting. Specifically, matrix density^18, 19^, ligand density,^20^ and matrix stiffness^21, 22^ have been suggested to be important parameters influencing angiogenic invasion. However, because these matrix properties are intrinsically coupled in natural ECMs, it is difficult to isolate the relative contribution of any one of these factors. Using a synthetic hydrogel to tune these properties orthogonally, we found that matrix crosslinking plays a critical role in modulating the extent, morphology, and even multicellularity of cell invasion. Crosslinking of synthetic gels has classically been used to vary the stiffness of a 2D substrate on which cells are seeded; this stiffness has been shown to dramatically impact cell spreading, proliferation, migration and differentiation^13, 14^. However, in 3D settings such as those investigated here, the degree of crosslinking alters not only matrix stiffness but also its degradability—the rate at which cells can carve space out of the matrix. Degradability has previously been shown to affect the ability of fully encapsulated single cells to spread into the matrix^23, 24^. By tuning degradability independently from matrix stiffness, we reveal here that ECM degradability is a key regulator of the collective nature of multicellular invasion. Multicellular strand-like cell migration is critical to not only the formation of functional blood vessels, but also to the creation of other numerous developmental structures. In addition, it is a fundamental process co-opted by disease processes, for example during cancer metastasis^25, 26^.

A hydrogel system where shape and dimensions can be defined upon initial crosslinking without subsequent swelling should have broad utility. While defined feature sizes and geometries have many potential uses, we have provided some examples in geometrically defined microgels, anatomically shaped tissue engineered constructs, and the incorporation of hydrogels into microfluidic devices. Some other classes of gels such as those based on natural components like collagen and fibrin are also non-swelling and could potentially be used for molding applications. However, their fibrous nature renders them structurally and mechanically more complex and further, their properties are more difficult to modulate orthogonally. Thus, such gels are difficult to use in studies that require control over individual matrix properties. Recently, several alternative strategies designing non-swelling hydrogels have been introduced including the use of lower polymer content^27^ or hydrophilic and thermoresponsive polymer building blocks exhibiting swelling and shrinking properties that oppose each other^5^. However, since these systems are based on tetra-armed poly(ethylene glycol) units with no additional reactive groups along the polymer backbone, it is more challenging to modify these hydrogels with cell adhesive functionalities without having to adjust conditions to maintain constant crosslinking. In our approach, we make use of a sugar-based polymer with ample reactive groups per repeat unit to independently tune hydrogel swelling, matrix mechanics, degradability, and ligand density. The concept of tuning hydrophobicity to modulate swelling could in principle be extended to many other polymer systems, enabling a wide range of applications where swelling has historically been a limitation.

## Methods

### Reagents

All reagents were purchased from Sigma Aldrich and used as received, unless otherwise stated.

### Synthesis of DexMA

Dextran (MP Biomedicals, MW 86,000 Da) was modified with methacrylate groups, as previously described^6^. In brief, dextran (20 g) and 4-dimethylaminopyridine (2 g) were dissolved in 100 mL anhydrous dimethyl sulfoxide, and varying amounts of glycidyl methacrylate (GMA) were added under vigorous stirring. The mixture was heated to 45 °C and allowed to react for 24 h. The solution was then cooled on ice and precipitated into 1 L ice-cold 2-propanol. The crude product was recovered by centrifugation, re-dissolved in milli-Q water and dialyzed against milli-Q water for 3 days with two solvent exchanges daily. Finally, the solution was lyophilized to obtain the pure product, which was characterized by ^1^H NMR spectroscopy in D_2_O. The degree of functionalization was calculated as the ratio of the proton integral (6.174 and 5.713 ppm) and the anomeric proton of the glycopyranosyl ring (5.166 and 4.923 ppm). As the signal of the anomeric proton of α-1,3 linkages (5.166 ppm) partially overlaps with other protons, a pre-determined ratio of 4% α-1,3 linkages was assumed and the total anomeric proton integral was calculated solely based on the integral at 4.923 ppm. A methacrylate/dextran repeat unit ratio of 0.7 was determined.

The input amount of GMA determined the resultant degree of dextran methacrylation. The formulation used to fabricate hydrogels for all cell studies was functionalized with 1.5 molar equivalents (relative to dextran) GMA, resulting in an average of one methacrylate on 71% of all repeat units.

### Preparation of non-cleavable DexMA hydrogels

For swelling studies, DexMA was dissolved at 10% w/v in PBS. 100 mg/mL Irgacure 2959 in ethanol was added to a final concentration of 0.2%. Solutions were mixed and photo-polymerized in poly(dimethylsiloxane) (PDMS, Sylgard 184, Dow Corning) molds using an Omnicure S2000 UV lamp (Exfo, Ontario, Canada) at 100 mW/cm^2^ (measured at 365 nm). The molds were removed, and the gels were allowed to swell in PBS for at least 24 h. Food coloring was added to the solution to better visualize the gel outlines for quantification of swelling.

For cell studies, a mixture of DexMA (18% methacrylation, 6.3% w/v) and cyclo[RGDfK(C)] (cRGD, Peptides International) (0.55 mM) was prepared in M199 media (Gibco) containing sodium bicarbonate (3.5% w/v) and HEPES (10 mM). The pH was adjusted to 8 with 1 M sodium hydroxide (NaOH) solution, initiating the Michael addition reaction between methacrylate and cRGD cysteine functionalities. After 30 min, the solution was neutralized with 1 M HCl and Irgacure 2959 in ethanol was added to a final concentration of 0.02%. Precursor solutions were spread onto glass coverslips and photo-polymerized at 20 mW/cm^2^ under argon for varying durations. Exposure times of 30, 40, 50, and 60 s yielded hydrogels with Young’s moduli of 0.5, 1.5, 4.4, and 8.9 kPa, respectively.

### Preparation of PEG and HA hydrogels

Poly(ethylene glycol) diacrylate (PEGDA, MW 6000 Da) was synthesized and hydrogels were photopolymerized following a previously published procedure^28^. Hyaluronic acid (HA) hydrogels were prepared from methacrylated HA with a degree of functionalization of 40% (relative to the number of repeat units) following a literature protocol^29^.

### Preparation of MMP-cleavable DexMA hydrogels

The cell adhesive peptide CGRGDS and the bis-cysteine crosslinker peptides CGPQGIAGQGCR (NCD) and CGPQGPAGQGCR (LD) were custom synthesized by Aapptec and supplied as trifluoroacetate salts at >95% purity.

A solution of DexMA (71% methacrylation, 4.4% w/v) and CGRGDS (3 mM) was prepared in M199 media containing sodium bicarbonate (3.5% w/v) and HEPES (10 mM). The pH was adjusted to 8 with 1 M NaOH to couple CGRGDS to DexMA. After 30 min, varying amounts (17–44 mM) of crosslinker peptide NCD or LD were added, the pH was re-adjusted to 8, initiating the hydrogel formation. Concentrations of crosslinkers determined the stiffness of the hydrogel (Fig. 3d). Hydrogels were allowed to polymerize for 1 h.

For 3D cell encapsulation, cells were added to the gel precursor solution at 0.5 × 10^6^ directly after the second pH neutralization step. Drops of the solution were added onto glass coverslips and the samples were polymerized under ambient conditions for 1 h. Gels were cultured in media, as described below.

### Mechanical testing

To determine the Young’s modulus of DexMA hydrogels, nanoindentation testing was performed with an atomic force microscope (MFP-3D, Asylum Research, Santa Barbara, CA) using a silicon-nitride tip (0.06 N/m) loaded with a 25 µm diameter polystyrene microsphere. Young’s modulus was determined by fitting force-indentation curves to established models for Hertzian contact of a spherical indentor on an elastic half space, assuming a Poisson ratio of 0.5.

### Angiogenic device fabrication

Angiogenic devices were fabricated according to a previously published procedure^12^. In brief, two patterned layers of PDMS, molded from photolithographically generated silicon masters, were bonded to each other and sealed against a glass coverslip to form the device housing. Two 400 μm diameter acupuncture needles (Hwato) were coated with 5 wt/vol% gelatin solution, cooled to 4 °C for 5 min, sterilized using UV and inserted into the device. An MMP-cleavable DexMA gel was cast inside the device, allowed to polymerize for 60 min and hydrated in PBS overnight. Devices were warmed to 37 °C for 1 h to melt away the gelatin coating prior to needle extraction. The gel was washed thoroughly with PBS and EGM-2 prior to cell seeding.

### Cell culture and experiments

Human foreskin fibroblasts (HFFs) were cultured in high-glucose Dulbecco’s Modified Eagle Medium (DMEM, Gibco) supplemented with 1% penicillin/streptomycin, l-glutamine and 10% fetal bovine serum. In a typical 2D experiment, DexMA hydrogels were seeded at 2000 cells/cm^2^.

Human umbilical cord vein endothelial cells (HUVECs, Lonza) were cultured in fully supplemented EGM-2 media (Lonza) and expanded to passage 4 prior to use in experiments. For angiogenic device experiments, HUVECs were seeded into one channel at 10^7^/mL and allowed to adhere to the bottom surface for 30 min. The device was inverted, cells were seeded to the top surface at 10^7^/mL, and allowed to adhere and spread for 2 h. Unattached cells were thoroughly washed out with EGM-2 and the devices were placed on a platform rocker (BenchRocker BR2000) to generate gravity-driven flow through both channels. Eight hours after seeding, an angiogenic growth factor cocktail consisting of 75 ng/mL VEGF (R&D Systems), 75 ng/mL MCP-1 (R&D Systems), 150 ng/mL PMA (Sigma), and 250 nM S1P (Cayman Chemical) was introduced to the second channel to induce angiogenic sprouting. MMP inhibitor Marimastat (Tocris Bioscience) was administered into both channels at 500 nM.

### Fluorescent staining and microscopy

HFFs on hydrogel samples were fixed with 4% PFA for 15 min (2D) or 1 h (3D) at room temperature. To visualize the organization of the actin cytoskeleton, cells were permeabilized with Triton X-100 for 5 min and stained with phalloidin-Alexa Fluor 488 (Life Technologies) for 1 h at room temperature. Nuclei were counterstained with DAPI.

HUVECs in devices were fixed with 3.7% glutaraldehyde for 30 min at room temperature. Samples were stained with phalloidin-Alexa Fluor 488 and DAPI overnight.

All samples were imaged at ×10 or ×40 on a Zeiss 200 M with a spinning disk head (Yokogawa CSU-10 with Borealis), environmental chamber, four laser lines, and photometric Evolve EMCCD camera. Images are presented as maximum intensity projections. Cell area was determined with a custom Matlab script. Sprout multicellularity was analyzed manually by counting the number of nuclei per sprout structure. Sprouts with more than six nuclei were defined as multicellular, and presented relative to the total number of sprouts. Cell density was determined from the total number of nuclei and the volume of gel containing sprout structures within each confocal stack.

### Hydraulic permeability

The hydraulic permeability of dextran gels was measured as previously described^30^. Briefly, gels were formed in microfluidic channels and a hydrostatic pressure gradient was established across the gel. By measuring the volumetric flow rate through the gel, the hydraulic permeability was computed using Darcy’s Law.

### Diffusivity

Diffusion coefficients were determined by introducing fluorescently tagged 3, 10, and 70 kDa dextran into 160 μm channels formed through dextran gels as previously described^12^. Time-lapse fluorescence microscopy was used to image the labeled dextran as it diffused into the hydrogels. The fluorescence intensity in the hydrogel was measured as a function of time, and the resulting profile was fit to the 1D unsteady solution of Fick’s second law of diffusion of dilute species^31^.

### Poisson’s ratio

To determine the Poisson ratio, cylindrical DexMA gels (6 mm diameter, 5 mm height) were cast in a PDMS mold and allowed to swell in PBS overnight. A micrometer-driven indenter was used to apply compressive axial strains (*E*_z_) while imaging the gel from the side to quantify transverse strain (*E*_xy_). The Poisson ratio was estimated for small strains (<5%) as −*E*_xy_/*E*_z_.

### Rheology

Shear (Gʹ) and loss (Gʺ) moduli of DexMA gels were measured using an AR-G2 rheometer (TA Instruments, New Castle, DE), equipped with a solvent trap and a 20 mm stainless steel plate geometry. Gel samples were prepared using identical reagents and methods as in the 3D angiogenic sprouting experiments. Once the geometry made firm contact with the samples, frequency sweeps from 0.1 to 10 Hz at 1% strain were performed, followed by strain sweeps between 0.1 and 50% at 1 Hz. Data were collected from multiple measurements of three independent samples.

### Statistics

Statistical differences were determined by ANOVA or Student’s *t*-test where appropriate, with significance indicated by *P* < 0.05. Sample size is indicated within corresponding figure legends. All data are presented as a mean ± standard deviation.

Each study was repeated three times. For experiments involving single-cell analysis, *n* ≥ 50 cells, for sprouting experiments, *n* ≥ 4 fields of view and for mechanical characterization, *n* ≥ 8 positions were analyzed.

### Data availability

The data sets generated and analyzed during the current study are available from the corresponding authors upon reasonable request.

## Electronic supplementary material

Supplementary Information
