# Supplementary material for: Matrix degradability controls multicellularity of 3D cell migration
Source: Nat Commun. 2017 Aug 29;8:371. doi: 10.1038/s41467-017-00418-6 (PMC5575316; doi:10.1038/s41467-017-00418-6)
Supplement: Supplementary file 1 — Supplementary Information [file 41467_2017_418_MOESM1_ESM.pdf]

Description of Supplementary Files

File Name: Supplementary Information
Description: Supplementary Figures and Supplementary Table

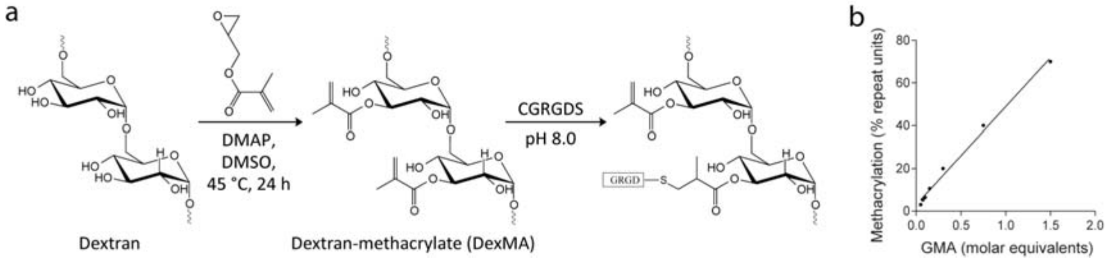

Supplementary Figure 1. Tunable methacrylation of dextran to control hydrogel backbone hydrophobicity. a, Dextran is reacted with glycidyl methacrylate (GMA) to generate methacrylated dextran (DexMA), subsequently functionalized with RGD to enable cell adhesion. **b**, Degree of methacrylation (in % repeat units) as a function of GMA concentration (in molar equivalents relative to dextran repeat units).

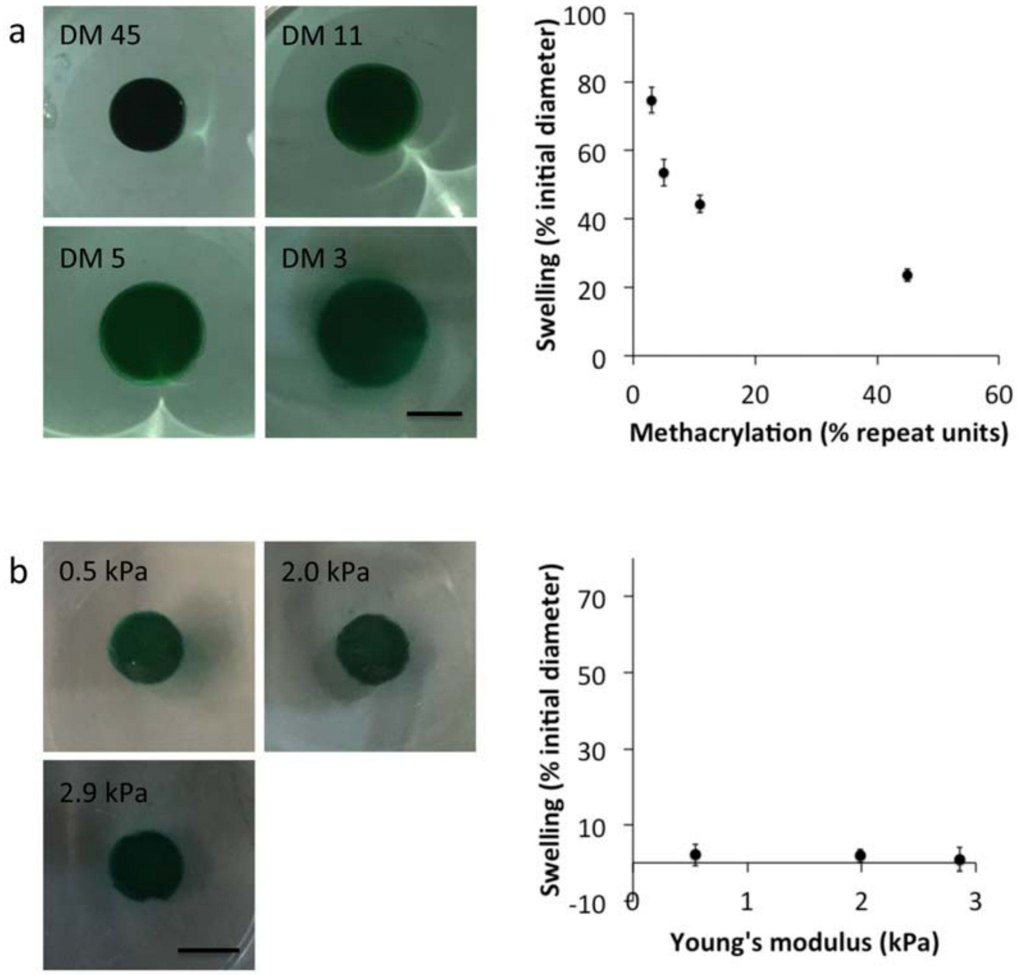

Supplementary Figure 2. Swelling of MMP cleavable DexMA hydrogels. a, Hydrogel swelling (defined as % of initial diameter) as a function of methacrylation (label indicates % methacrylation) after equilibrium swelling. **b,** Swelling as a function of the mechanical properties (Young's modulus in kPa) of the gels. All data is presented as a mean ± s.d. (scale bars 0.5 cm).

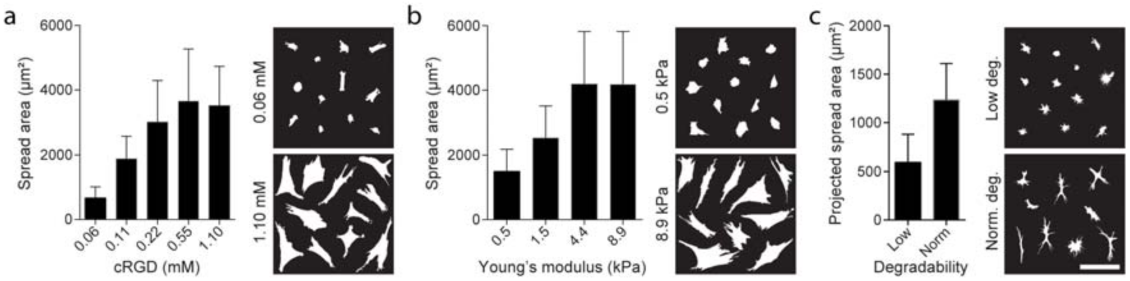

Supplementary Figure 3. Biofunctionalization of DexMA hydrogels. a, Quantification of human foreskin fibroblast (HFF) spread area (left) and representative cell outlines (right) as a function of cRGD concentration (left). **b**, Quantification of HFF spread area (left) and representative cell outlines (right) as a function of the Young's modulus of hydrogels (left). **c**, Quantification of HFF spread area (left) and representative cell outlines (right) for different crosslinker peptide degradabilities (native collagen CGPQGIAGQGCR versus low degradability CGPQGPAGQGCR sequence). Scale bar: 100 µm. All data is presented as a mean ± s.d.

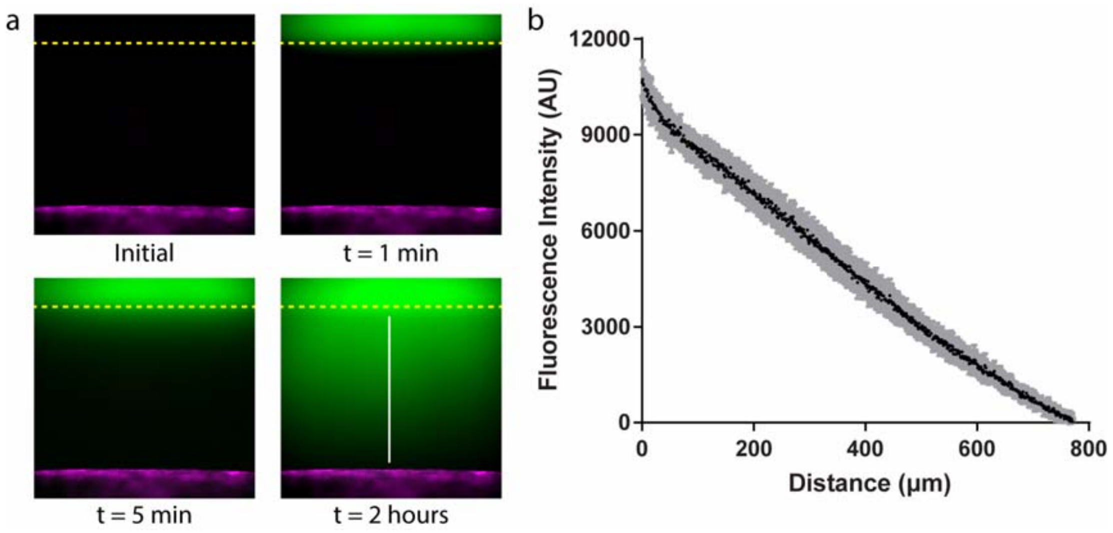

Supplementary Figure 4. Characterization of the gradient formed inside the angiogenic microfluidic device. a, Fluorescently labeled dextran (80 kDa MW) was added to the source channel and allowed to diffuse towards the sink channel. **b,** Intensity profile of the resulting gradient of fluorescently labeled dextran (white line in bottom right panel of **a** indicates measurement location).

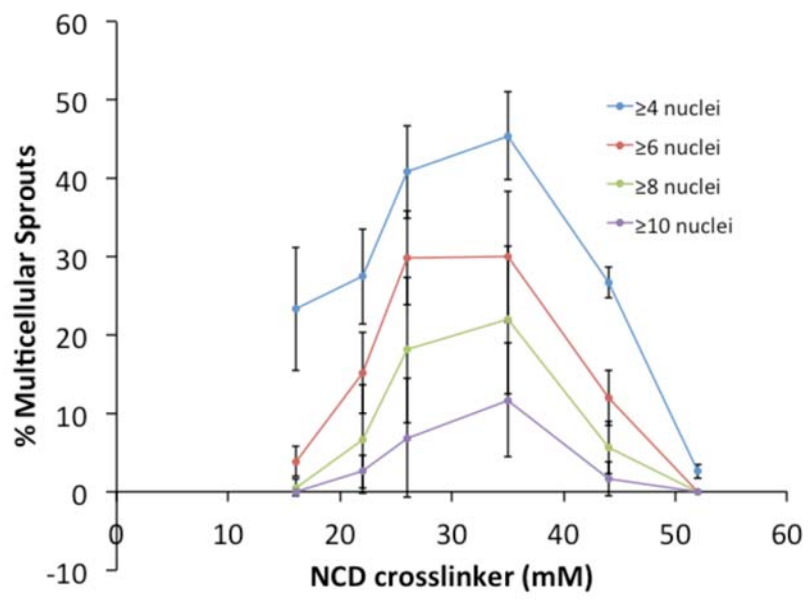

Supplementary Figure 5. Analysis of sprout multicellularity with different nuclear cutoffs. Sprout multicellularity was determined by counting the percentage of actin structures containing above a preset cutoff number of nuclei. To examine the effect of this cutoff value's selection, data from the study screening NCD crosslinker concentration was reanalyzed using different a range of values (4, 6, 8, and 10 nuclei).

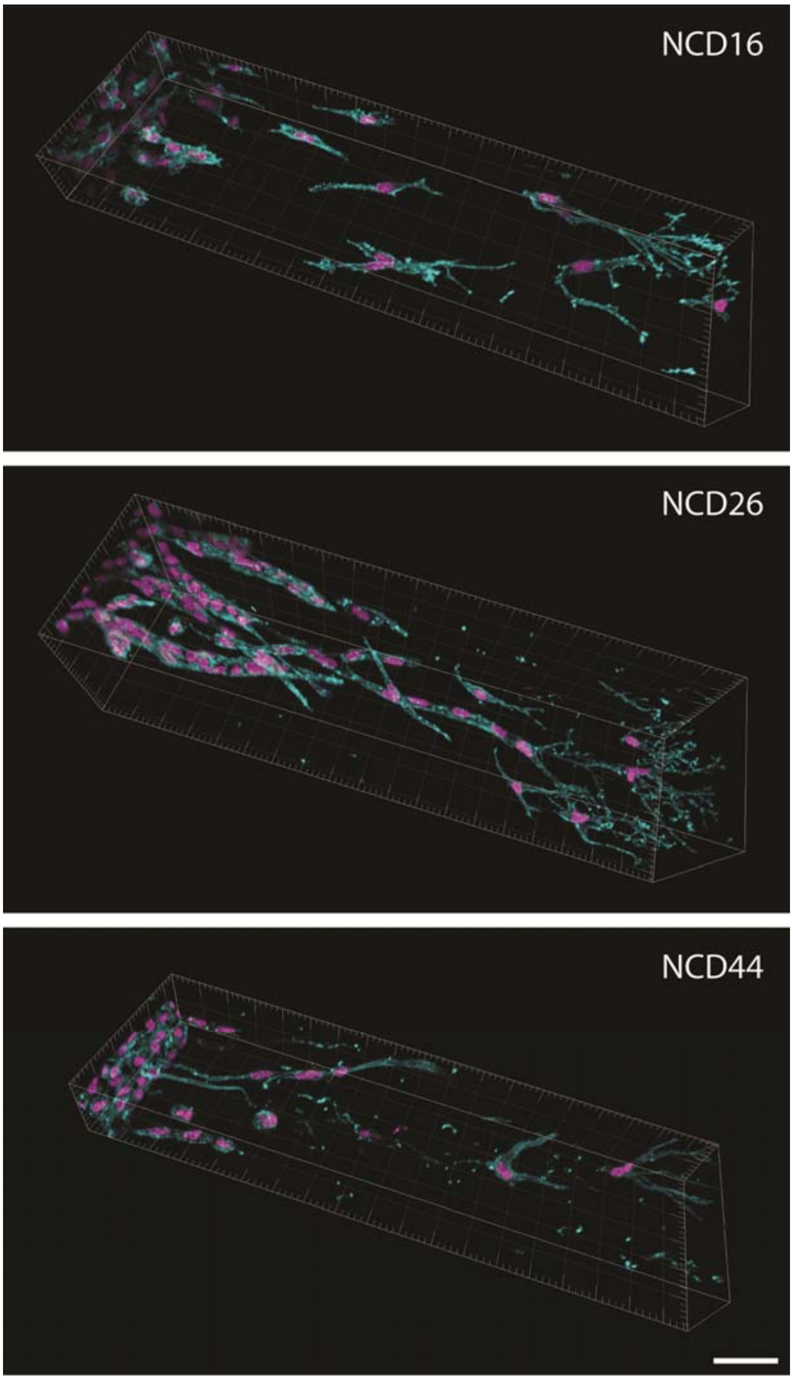

Supplementary Figure 6. Three-dimensional reconstructions of angiogenic sprouts at different NCD crosslinker concentrations. Reconstructions were generated in Imaris (scale bar 200 μm).

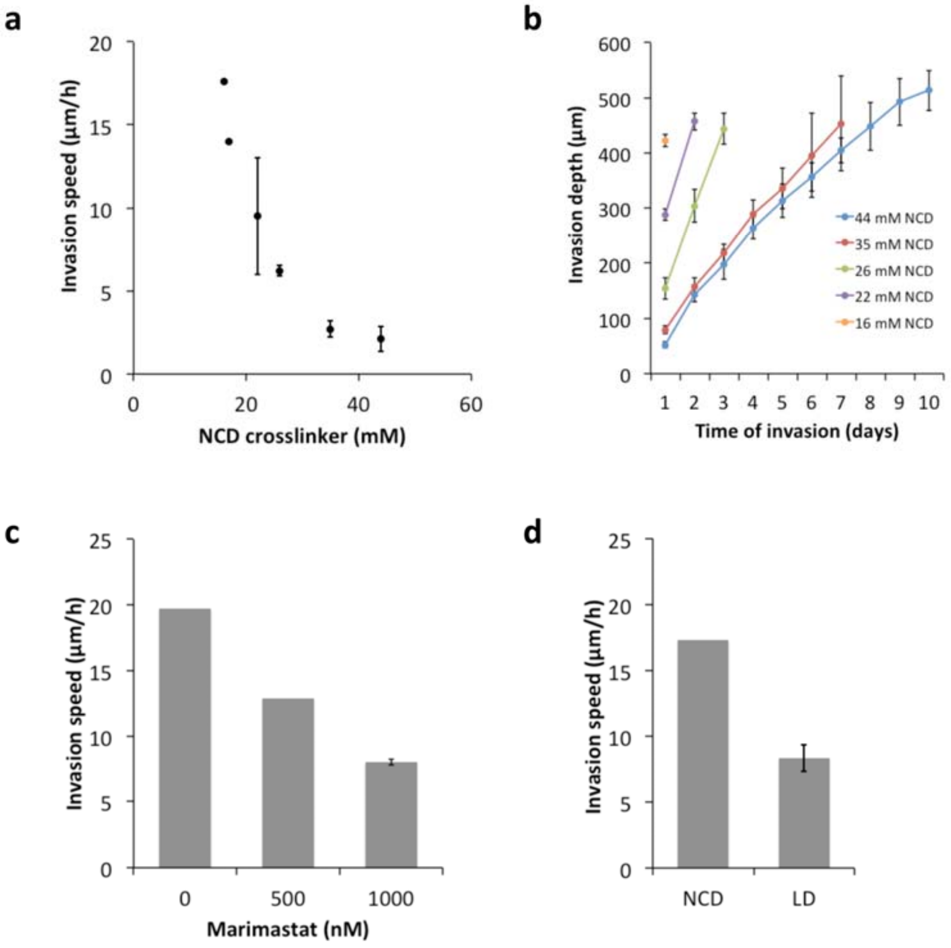

Supplementary Figure 7. Speed of cell invasion from endothelialized channels into 3D MMP cleavable DexMA hydrogels. Average invasion speed as a function of (**a**) NCD crosslinker concentration, (**c**) broad spectrum MMP inhibitor Marimastat concentration and (**d**) hydrogel degradability. **b,** Daily time-course of endothelial cell invasion depth for hydrogels with varying NCD crosslinker concentration.

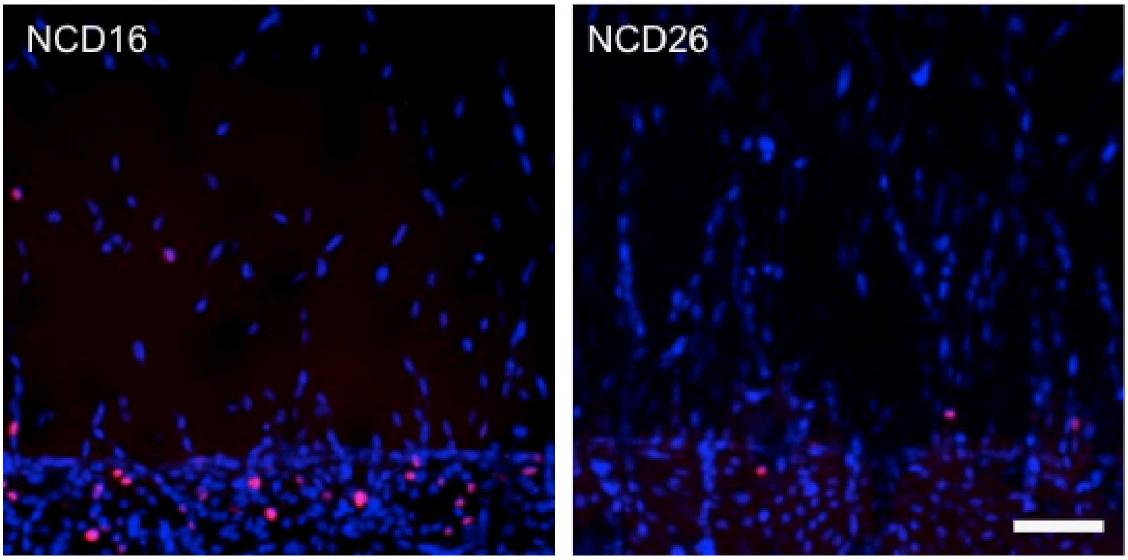

Supplementary Figure 8. Proliferation of HUVECs invading into DexMA hydrogels of different NCD crosslinker concentrations. Proliferation was assayed by EdU incorporation (EdU was added to the cell culture media for 48 h prior to fixation). Composite fluorescence images stained for EdU (red) and DNA (blue) (scale bar 50 μm).

	Diffusivity			Permeability	Poisson's ratio
	**3 kDa**	**10 kDa**	**70 kDa**		
NCD16	2.32×10^{-11} $\pm 1.31\times10^{-11}$	9.62×10^{-12} $\pm 2.92\times10^{-12}$	1.12×10^{-12} $\pm 0.76\times10^{-12}$	3.12×10^{-15} $\pm 1.31\times10^{-15}$	
NCD26	3.37×10^{-11} $\pm 1.11\times10^{-11}$	8.39×10^{-12} $\pm 1.86\times10^{-12}$	1.34×10^{-12} $\pm 0.38\times10^{-12}$	2.86×10^{-15} $\pm 1.38\times10^{-15}$	0.44 $\pm$ 0.02

Supplementary table 1. Characterization of diffusivity, hydraulic permeability and Poisson's ratio of MMP cleavable hydrogels.
